# Leptospirosis in an asplenic patient -case report

**DOI:** 10.1186/s12879-020-4869-3

**Published:** 2020-02-28

**Authors:** J. García-Méndez, E. Cervera-Ceballos, D. Atilano-López, S. Arroyo-Escalante, D. Moncada-Barrón, M. Leyva-Leyva, R. Hernández-Castro, E. M. Carrillo-Casas

**Affiliations:** 1 Dirección de Docencia, Instituto Nacional de Cancerología, Mexico City, Mexico; 2 Departamento de Microbiología y Parasitología, Facultad de Medicina, UNAM, Mexico City, Mexico; 3 Laboratorio de Diagnóstico-Bacteriología, Sección Leptospira, Facultad de Medicina Veterinaria y Zootecnia, Mexico City, Mexico; 4 División de Laboratorio Clínico, Hospital General “Dr. Manuel Gea González”, Mexico City, Mexico; 5 Departamento de Biología Molecular e Histocompatibilidad, Dirección de investigación, Hospital General “Dr. Manuel Gea González”, Mexico City, Mexico; 6 Departamento de Ecología de Agentes Patógenos, Dirección de investigación, Hospital General “Dr. Manuel Gea González”, Mexico City, Mexico

**Keywords:** Leptospirosis, *Leptospira*, Weil’s disease, Serovar Bratislava, Idiopathic thrombocytopenic purpura, Immunosuppression, Chronic myeloid leukemia, Splenectomy

## Abstract

**Background:**

The presentation of clinical leptospirosis has been historically associated with animal workers, slaughterhouse workers and medical veterinarians. This association has shifted to be related to flooding events and outdoor activities; few cases are related to high-risk factors found in immunosuppressed patients. Scarcely a handful of cases have serological evidence of immune response against *Leptospira* serovar Bratislava representing serogroup Australis, a serovar associated with poor reproductive performance in swine and horses, and recently with cats.

**Case presentation:**

Herein, we describe a rare clinical presentation of disseminated *Leptospira* infection in an immunosuppressed 65-year-old woman. She was admitted to the emergency room with fever, bacteraemia, bilateral uveitis and pulmonary involvement. The patient denied outdoor activities; she only had wide exposure to faeces and urine from cats living in her home. Her medical history included idiopathic thrombocytopenic purpura (ITP) diagnosed at the age of 18. She did not respond to medical treatment, and a splenectomy was performed. At age 60, she was diagnosed with Chronic Myeloid Leukemia (CML), and was treated with a tyrosine kinase inhibitor (TKI) –Imatinib. The patient voluntarily discontinued the treatment for the last 6 months. After extensive workup, no microorganisms were identified by the commonly used stains in microbiology. The diagnosis was performed through dark-field microscopy, microagglutination test (MAT), *Leptospira* genus-specific PCR, the *IS*1500 PCR for identification of pathogenic species, and 16*S* based sequencing for the genus identification.

**Conclusion:**

Immunosuppressed patients may acquire uncommon infections from ubiquitous microorganisms. In this case, serology evidence of exposure to *Leptospira* serovar Bratislava by MAT and the presence of the *Leptospira* genus were identified. It should be on mind for the diagnosis in otherwise healthy patients, and thoroughly search on splenectomised patients exposed to animals. Additionally, this report highlights the usefulness of PCR for diagnosis of this potentially life-threatening illness.

## Background

Leptospirosis is a worldwide zoonosis caused by pathogenic *Leptospira.* The global incidence of severe leptospirosis is estimated to be more than 1 million per year, with a fatality rate ranging around 10%. In the past, the disease was related to professionals of rural areas and the flooding season. Due to climate change conditions, leptospirosis is an increasing public health issue in many developing countries [1, 2]. Currently, its presentation in urbanised areas is related to outdoor activities and animal contact. Mexico is considered a country of moderate incidence [3]; it has geographic regions with the environmental conditions of temperature and precipitation favourable for *Leptospira* infection all along the year such as the states of Tabasco and Quintana Roo [4, 5].

The clinical presentation of acute leptospirosis is typically a rapid onset, febrile, systemic disease. The signs are mild and self-limiting; the leptospiremic (initial) phase includes high spiking fever, headache, conjunctivitis, and myalgias, which last 4 to 9 days. There is no pathognomonic clinical feature; however, acute onset and high spike fever indicate the clinician to consider leptospirosis among the differential diagnosis and the medical history that points out a probable exposure to *Leptospira*. The severe presentation of leptospirosis, in addition to the above symptoms, also includes jaundice, haemorrhage, and acute renal failure. Unusual manifestations include pulmonary, cardiovascular, neurological, gastrointestinal, ocular and other systemic symptoms [6, 7].

Leptospirosis in an asplenic patient is rare and has not been reported previously. Herein we describe a leptospirosis case due to a cats and swine related *Leptospira* serovar in an asplenic patient, who had a history of non-responsive IPT and under chronic phase of CML.

## Case presentation

**Fig. 1:**
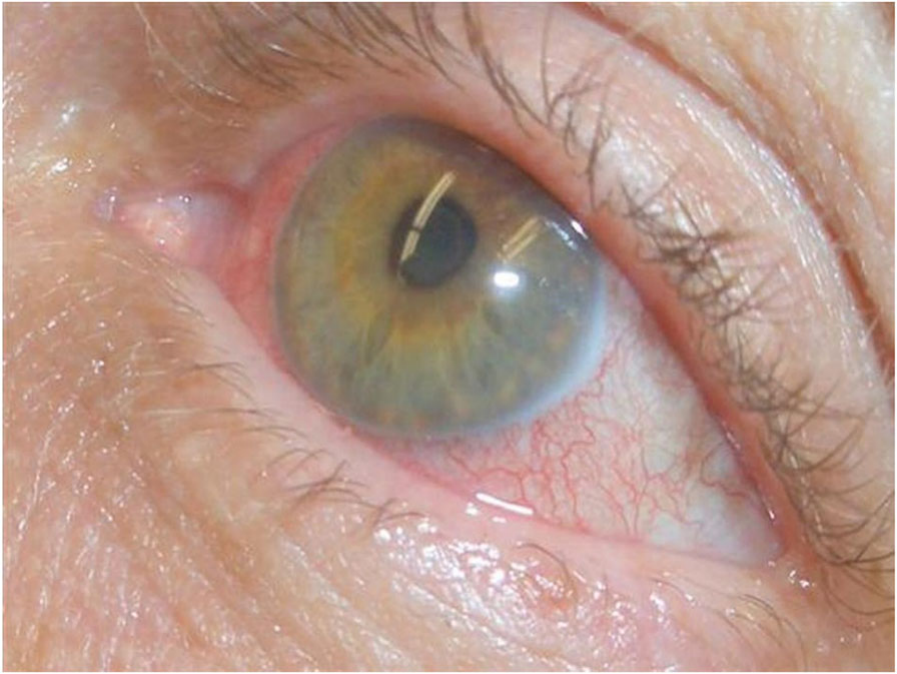
Clinical view of the inflammatory process in the patient’s left eye

A 65-year-old female was admitted to the emergency room. The patient had a medical history of idiopathic thrombocytopenic purpura (ITP) diagnosed at the age of 18, refractory to steroid-based treatment. A splenectomy was performed, and she remained with normal platelet counts. She was diagnosed at 60 years-old with chronic myeloid leukaemia (CML), and received treatment with tyrosine kinase inhibitor (TKI)-Imatinib. The diagnosis was confirmed by bone marrow aspiration and fluorescent in situ hybridisation (FISH), in which 22% were negative cells and 78% positive cells for the Philadelphia chromosome. The qPCR for BCR-ABL/ABL was 40.22% (15,420.24 ABL copies, and 6202.4 BCR-ABL copies). On admission, she admitted to voluntarily had stopped the Imatinib treatment 6 months before due to a major depression, had fever and worsening eyesight (later diagnosed as acute uveitis) (Fig. 1). She had no history of travelling to leptospirosis endemic areas, nor alcohol, drug or tobacco abuse, neither other medications intake. Nevertheless, she had close contact with her pet cat. She was admitted to the hospital with acute respiratory failure, bacteraemia and uveitis.

**Fig. 2:**
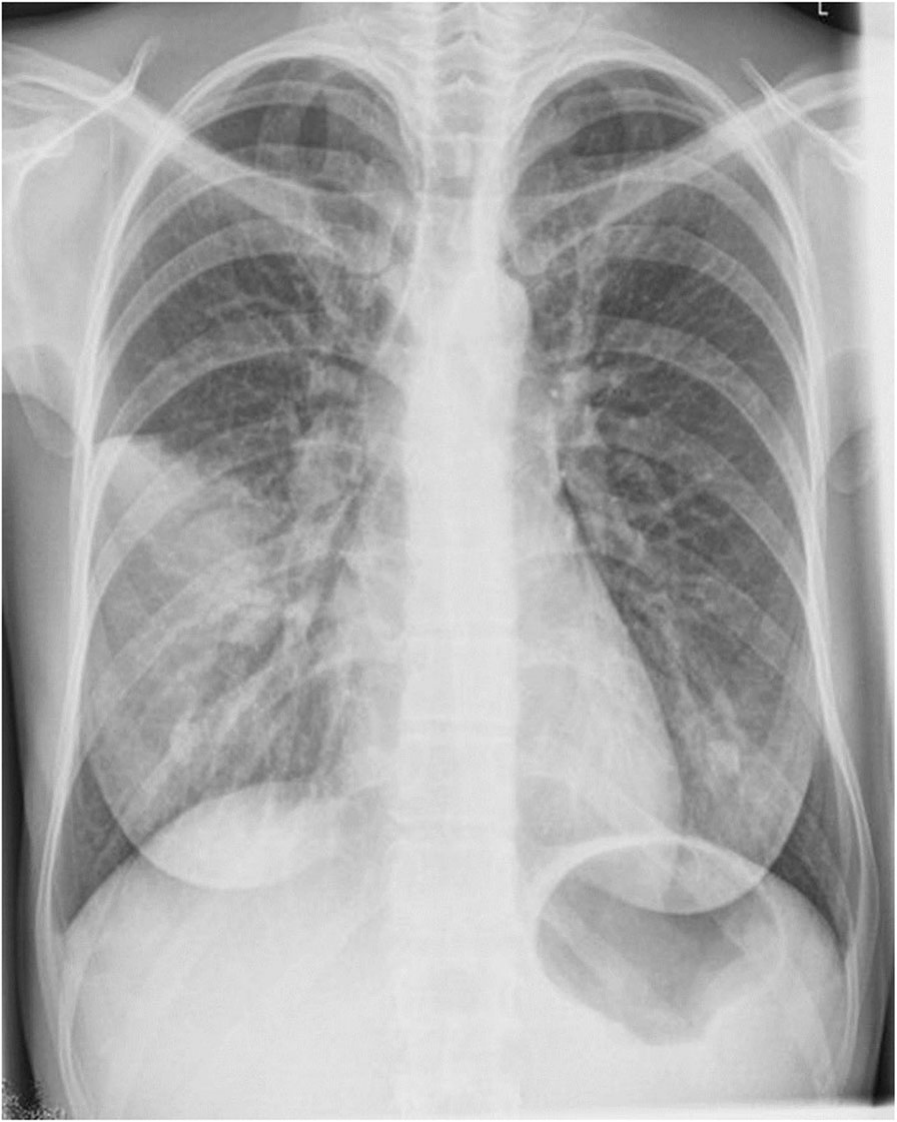
In the chest x-ray, the lung had alveolar occupation of the right middle lobe due to an atypical pneumonia

Laboratory tests showed leukocyte count of 144.6 × 10^3^/ml, hemoglobin 12.4 g/dl, platelets 763 × 10^3^/ml, glucose level of 116 mg/dl, and albumin level of 3.0 mg/dl. Liver function test showed malnourishment and low protein level. Renal function test showed high creatinine level indicating an acute kidney injury mainly associated with the inflammatory response, and electrolytes were within normal limits. Additionally, the levels of C-reactive protein and procalcitonin were moderately high (Supplementary Table 1). Due to respiratory deterioration, a sputum culture was performed, and *E. coli* was isolated. In México, at the time the patient was treated, there was an outbreak of influenza, reason why a diagnostic qPCR for the influenza virus was performed; the result was negative. The chest X-Ray revealed multiple pneumonia foci (Fig. 2). She was started on empirical broad antibiotic regimen. Blood and urine cultures collected on admission showed no microorganisms grown from conventional medium or seen on Gram, Ziehl-Nielsen, and Giemsa stains. The blood cultures in the BacT/ALERT® FA medium (bioMérieux, Durham NC), after 2 days of incubation at 34 °C, were positive. Unstained spirochaete-like microorganisms were observed by dark-field microscopy. Due to clinical and microbiological finding, the antimicrobial treatment was changed to penicillin 20 million UI/ IV, plus doxycycline 100 mg twice a day. Subsequently, two blood samples were tested by Microagglutination test (MAT) separated from each other by 10 days (Supplementary Methodology Document). Differential diagnosis were performed, and negative results were reported. After the successful treatment, the CML treatment was resumed with a second-generation TKI (Dasatinib). A year later, the FISH analysis showed 35 negative cells and 65 positive cells for the Philadelphia chromosome. The aspiration of bone marrow showed an adequate maturation, compatible with CML in chronic phase.

### Microagglutination test (MAT)

Microagglutination test (MAT) was performed as previously described by the Pan American Health Organization [8]. The patient’s sera were diluted to 1/50 for a screening test and 50 μl of live cultures of 4 to 7 days in EMJH medium of twelve *L. interrogans* serovars were used as antigens (Autumnalis, Bataviae, Bratislava, Canicola, Celledoni, Grippotyphosa, Hardjoprajitno, Icterohaemorrhagiae, Pomona, Pyrogenes, Tarassovi, Wolffi and Mini) (Supplementary Table 2). Each serovar was added into a column of a 96-well flat end microtitre plate (Nunc, Maryland, USA); a negative control was included for each serovar. The plate was gently stirred and incubated 1 hour at room temperature. Reading was performed in dark-field microscopy (Carl Zeiss, Germany). The patient’s sera were serially diluted from 1/25 to 1/1600, and the microagglutination was observed for each serovar. The final titre represents the maximum dilution in which the agglutination grade two was noted.

### Leptospira isolation

From the patient’s blood sample, three drops were seeded into EMJH liquid medium and maintained at 30 °C. Cultures were periodically observed in dark-field microscopy to spot spirochaetal forms and maintained for 6 months before being considered negative cultures. In parallel, the patient’s blood sample was inoculated to a Syrian golden hamster (*Mesocricetus auratus*) for bacterial isolation [9] (Supplementary Methodology Document and Supplementary Table 3).

### DNA extraction

DNA was extracted from the patient’s blood samples and the hamster’s tissues with the DNeasy Blood &Tissue Kit (QIAGEN, Cal, USA), according to the manufacturer’s instructions and suspended in 50 μL of nuclease-free water. DNA was quantified using an Epoch microplate spectrophotometer (Biotech) and stored at 4 °C.

### PCR

The *Leptospira* genus-specific PCR based on the *23S* rDNA was used to amplify a 482 bp fragment [10]. A second PCR was performed to identify only pathogenic strains based on the amplification of the insertion sequence IS*1500* of *L. interrogans* (sensu *lato*) [11] (Supplementary Methodology Document). The electrophoresis of the amplified products were stained with ethidium bromide on 1.6% agarose gels and visualised. Further genus identification was performed based on sequencing of the 16*S* rRNA gene, using the primers 27f (5′-AGAGTTTGATCMTGGCTCAG-3′) and 1492r (5′-TACGCYTACCTTGTTACGACTT-3′) with DNA extracted from the first blood sample as template [12]. A PCR product of 1432 bp was amplified, purified, and DNA was sequenced in both directions. Nucleotide sequence was determined with Taq FS Dye Terminator Cycle Sequencing Fluorescence-Based Sequencing, and analysed on an Applied Biosystems 3730 DNA sequencing system (Foster City, CA, USA). The sequence was registered in Genbank under the accession number MN545905. It has 100% homology with *Leptospira interrogans* strains (CP011410.1, AE016823.1, AE010300.2, and CP018146.1) and variable homology with reference sequences (Supplementary Methodology Document and Supplementary Table 4).

In summary, the results support the diagnosis of leptospirosis; abundant spirochaetal forms, at least 10^6^ *Leptospires*/mL were observed in dark field microscopy, The MAT results (Supplementary Table 2) indicated the serovar Bratislava as the most probable causing serovar because the patient’s serum reacted with Bratislava (Jez Bratislava) in titres of 1/320. This titre was the highest in the first sample and stayed in a steady-state in the second sample. There was no other serovar with titres at this level. Titres to Serovars Bataviae (Van Tienen); Canicola (Hond Utech IV); Grippotyphosa (Moska V); Hardjo (Hardjoprajitno), and Pomona (Pomona) were lower than 1/80 in both samples, and those for Pyrogenes (Salinem) fell from 1/160 to 1/80 in the second sample. During the *Leptospira* isolation attempt, its survival was sustained for about a month in the EMJH medium, but it did not flourish. The PCRs based on the23*S* rDNA and the *IS*1500 identified the presence of the bacteria DNA in the patient’s blood samples, in the hamster tissues (Supplementary Table 3), and the *Leptospira* genus was identified by the 16*S* based sequencing.

## Discussion and conclusions

Our patient was in the chronic phase of CML and voluntarily stopped TKI treatment losing the haematological response, which coincided with leptospirosis. The primary mechanism of immunosuppression related to the acquisition of the *Leptospira* infection was asplenic status. In theory, CML could also be associated with deregulation of the immune system. However, we are aware that there is no strong clinical evidence to support it.

Mild Leptospirosis cases escape diagnosis in immunosuppressed patients due to HIV (Human Immunodeficiency Virus) or other conditions [13]. To the best of our knowledge, there are no reports of leptospirosis in splenectomised patients. Splenectomy is a second-line treatment for ITP when previous therapeutic measures had failed. The procedure is not strictly “curative” because the immune mechanism persists, and the consequence is that the patient has a permanent immunosuppression condition [14]. Therefore, splenectomy is associated with an increase of overwhelming post-splenectomy infections (OPSI), defined as infections that require admission to intensive care unit as a late complication following splenectomy [15]. OPSI are caused by unusual encapsulated bacteria, including *Streptococcal pneumonia, Haemophilus influenzae, Neisseria meningitidis*, and other organisms such as *Capnocytophaga canimorsus* [16–18], and *Cryptococcus neoformans* [19]. The risk of OPSI is minimised by the administration of a broad vaccination scheme. In Mexico, the *Leptospira* preventive vaccination in humans has not been approved. Therefore, the patient’s vaccination scheme did not include it.

The asplenic status is a specific risk factor that increase up to 2.2% the risk to develop pneumonia [20] According to guidelines, the pathogen isolated from blood cultures is the definite cause of the pneumonia except for the sputum isolation of *Legionella pneumophila, Mycobacterium tuberculosis, Pneumocystis carinii cysts* or *trophozoites* [21]. Otherwise, the isolation from sputum are presumptive, but must be confirmed by more than one sample with heavy to moderate growth. The diagnosis of *E. coli* pneumonia is based on radiographic evidence of bronchopneumonia of the lower lobes coupled with positive sputum and positive blood cultures for *E. coli* [22, 23]. To uphold the diagnosis of pneumonia due to *E. coli,* at least two of these criteria should be met, and be supported by clinical suspicion and the patient’s history. In this case, not all the criteria to diagnose *E. coli* pneumonia were met, and the *E. coli* sputum culture was considered a contamination due to oropharyngeal secretions [22].

Individuals subjected to splenectomy may form antibodies quite normally to antigens given subcutaneously but not respond well to antigens administered intravenously [24]. The asplenic status of the patient is relevant in the development of leptospirosis because the spleen participates in the removal of *Leptospira*, and various bloodborne pathogens as it produces opsonins that promote phagocytosis [25]. In the hamster model, *Leptospira* circulates in blood in the leptospiremic phase of the disease, and the histopathological changes that are produced in the spleen include cellular necrosis in the splenic cord, dilated sinusoids, congested hemorrhagic areas, and infiltration of inflammatory cells in the splenic parenchyma and sinusoids [26].

During the acute phase of illness, conjunctiva congestion, panuveitis with or without hypopyon are common clinical findings [27]. Therefore, it was not possible to differentiate whether the uveitis was a consequence of previous treatment or due to leptospirosis. In this immunosuppressed patient, not all haematological manifestations were due to leptospirosis, although some abnormalities may be associated with it [28].

Three radiographic patterns have been described in patients with pulmonary involvement during leptospiral infection; small nodular densities, diffuse ground-glass densities and rarely, confluent areas of consolidation [29]. In this case, pneumonic foci were observed yet only serovar Grippotyphosa, Valbuzzi, and Australis have been associated with pulmonary manifestations [30–32].

Serogroup Icterohaemorrhagiae is the most frequent in human infections, followed by Canicola, Grippotyphosa, Pyrogenes, Pomona and Australis, which may vary in each geographic region [33]. This patient was exposed somehow to serovar Bratislava, a globally distributed serovar but with unknown epidemiology [34]. This serovar is mostly associated with swine and cattle reproductive failure [35, 36], and maintained by dogs and horses [34]. This serovar is currently related to rural and domestic cats. Cats can be infected without clinical signs or seem clinically unapparent [37–39], needing an extended incubation period to develop the disease [40]. Cats may shed as much *Leptospira* as dogs do [41, 42]. Therefore, cats can be a source of urban leptospirosis [38, 42–44], and in this case, the patient’s cat may have been a potential infection source.

The Bact/ALERT® Microbial Detection System detects the microbial growth via the colourimetric detection of changes in the CO_2_ concentration [45], and the Bact/ALERT® FA media can support viable *Leptospira* up to 9 days [46, 47]_**.**_ In our laboratory experience, the negative results of the Gram, Ziehl-Nielsen, and Giemsa stains, coupled with the slight change in the CO_2_ level guided us to foresee the possibility of a slow-growing microorganism. The first blood sample was taken during the acute phase of leptospirosis, in which the patient was septicemic, reason why it was possible to visualise under dark-field microscopy.

Culture confirmation is the gold standard for clinical cases. We attempted *Leptospira* isolation during the leptospiremic phase in the hamster model, based on previous studies [9]. Nonetheless, *Leptospira* isolation and culture are difficult to be obtained; its persistence depends on the serovar [9, 48]. In particular, serovar Bratislava and close related strains are fastidious serovars [34]. Reason why our difficulties in achieving isolation are understood.

The MAT is a serogroup-specific test, its threshold titre is established according to the prevalence in each geographical region; for example, it is set at 1/100 for mainland France and 1/400 for endemic zones [33]. The official guidelines (NOM-029-SSA2–1999) establish the MAT threshold for humans at 1/80 in the first sample, confirmed by a second sample with the double or higher titres, or by the bacteria isolation or PCR. Titres under 1/80 are evidence of previous exposure to *Leptospira*, but not as the result of a current infection. The patient’s first sample showed a 1/320 titre to *L. interrogans* serovar Bratislava, and 1/160 to *L. interrogans* serovar Pyrogenes. The second sample showed titres as high as the previous sample to *L. interrogans* serovar Bratislava, low titres to *L. interrogans* serovar Hardjo and *L. interrogans* serovar Grippotyphosa. These MAT results are explained by two coexistence, the early and accurate treatment that halted leptospires quickly enough and the patient’s immunosuppression condition which are reflected as constant titres in the second sample, as observed by other researchers [49]. After treatment, and during the 10 months of follow up, MAT titres diminished, the uveitis improved, and she had no further leptospirosis signs.

Other diagnostic options include PCR amplification of bacterial DNA from blood during the first week after symptoms onset [33], qPCR applications [50–53], and sequence-based identification of *Leptospira* [54–56]. However, results should be validated by MAT because *Leptospira* is not always present in blood, as it could be removed if the treatment has begun. Additionally, MAT can be false negative considering that antibodies may rise until the second or third week of the disease [33]. In this case, the clinical judgment and suspicion was of paramount value to guide us to dark-field observation and the penicillin-based treatment [57], enhanced with doxycycline added along with intravenous fluids. Even though the unarguable conclusion of the infecting serovar cannot be drawn without isolation, the observations during blood culture and the MAT high titres to *L. Bratislava* gave us reasonable evidence of the *Leptospira* exposure.

In circumstances of atypical presentations of leptospirosis, diagnosis may be aided by PCR [58]. PCR detects the presence of nucleic acids of extremely low number of microorganisms; around two to ten cells [59, 60]. PCR cannot distinguish between viable and dead cells because all of them contribute to a positive signal. Even in culture-negative blood samples, PCR may be positive if the patient has received an effective antimicrobial drug but have not cleared nonviable organisms [61]. This condition happens in splenectomised patients, in which *Leptospira* may remain dead or alive for extended periods in comparison to immunocompetent patients, aside that they have inefficient antibody production. In this case, PCR confirmed the *Leptospira* genus as the aetiological agent and its circulation in the bloodstream, and ruled out a false positive diagnosis [62].

Finally, we want to emphasise the need of increased awareness in healthcare providers to consider leptospirosis in those cases in which infections may be reported as culture-negative severe infections [63], especially in asplenic patients, as this zoonosis may have an unusual presentation in immunosuppressed patients so that appropriate therapy can be initiated.

## Supplementary information

**Additional file 1: Supplementary. Supplementary Methodology document. Table 1.** Laboratory tests summary**. Supplementary Table 2.** Results of the Microscopic agglutination test (MAT). **Supplementary Table 3.** Results summary**. Supplementary Table 4**.- Results of BLAST with Reference Sequences.

## Data Availability

All data generated or analysed during this study are included in this published article and its supplementary information file.

## Abbreviations

CML: Chronic Myeloid Leukemia
EMJH: Ellinghausen-McCullough-Johnson-Harris
FISH: Fluorescent In Situ Hybridisation
ITP: Idiopathic Thrombocytopenic Purpura
MAT: Microagglutination test
OPSI: Overwhelming post-splenectomy infections
PCR: Polymerase Chain Reaction
