# Supplementary material for: Leptospirosis in an asplenic patient -case report
Source: BMC Infect Dis. 2020 Feb 28;20:186. doi: 10.1186/s12879-020-4869-3 (PMC7048021; doi:10.1186/s12879-020-4869-3)
Supplement: Supplementary file 1 — Additional file 1: Supplementary. Supplementary Methodology document. Table 1. Laboratory tests summary. Supplementary Table 2. Results of the Microscopic agglutination test (MAT). Supplementary Table 3. Results summary. Supplementary Table 4.- Results of BLAST with Reference Sequences. [file 12879_2020_4869_MOESM1_ESM.docx]

| Supplementary Table 1. Laboratory tests summary | | | | | | | | | | | | | | |  |  |
| --- | --- | --- | --- | --- | --- | --- | --- | --- | --- | --- | --- | --- | --- | --- | --- | --- |
|  | ANALISIS DATE | | | | | | | | | | | | | | units | Reference interval |
|  | 12/17  2012 | 12/19  2012 | 01/04  2013 | 02/18  2013 | 10/10  2013 | 10/11  2013 | 09/20  /2013 | 10/21  2013 | 10/24  2013 | 12/23  2013 | 02/19  2014 | 05/22  2014 | 12/09  2014 | 03/09  2015 |  |  |
| Erythrocytes | 3.09  ▼ | 3.19 | 3.08 ▼ | 4.52 | 4.59 | 3.87  ▼ | 5.24 | 3.98  ▼ | 3.96  ▼ | 3.48  ▼ | 3.96  ▼ | 3.82  ▼ | 3.72  ▼ | 5.18 | 10^6^/μL | 4.20 – 5.40 |
| Hematocrit | 33.5 ▼ | 33.9  ▼ | 32.9 ▼ | 43.2 | 40.3 | 33.9  ▼ | 46.1 | 34.6  ▼ | 33.9  ▼ | 31.7  ▼ | 35.1  ▼ | 38.6  ▼ | 33.3  ▼ | 41.2 | % | 39-51 |
| Hemoglobin | 10.6 ▼ | 10.9 ▼ | 10.5 ▼ | 14.2 | 12.4  ▼ | 10.3  ▼ | 13.5 | 10.8  ▼ | 10.8  ▼ | 10.1  ▼ | 11.1  ▼ | 12.6  ▼ | 10.7  ▼ | 12.9  ▼ | g/dL | 13.0-17.0 |
| Mean globular volume | 108.5 ▲ | 106.4▲ | 107.0▲ | 95.5 | 87.7 | 87.6 | 88.0 | 87.2 | 85.4 | 91.3 | 88.6 | 100.9  ▲ | 89.3 | 79.4  ▼ | fL | 80.0-99.0 |
| Average corpuscular hemoglobin | 34.3  ▲ | 34.1 ▲ | 34.1 ▲ | 31.4 | 27.1 | 26.7  ▼ | 25.7  ▼ | 27.1 | 27.1 | 29.0 | 28.0 | 33.0 | 28.6 | 25.0  ▼ | pg | 27.0-33.0 |
| Average concentration of hemoglobin | 31.6  ▼ | 32.0 | 31.9 ▼ | 32.8 | 30.9  ▼ | 30.5  ▼ | 29.3  ▼ | 31.1  ▼ | 31.8  ▼ | 31.7  ▼ | 31.5  ▼ | 32.7 | 32.1 | 31.4  ▼ | g/dL | 32.0 -37-0 |
| Erythrocyte distribution width | 18.5 ▲ | 18.6  ▲ | 24.9 ▲ | 20.2  ▲ | 18.6  ▲ | 18.3  ▲ | 19.0 ▲ | 18.0  ▲ | 17.1  ▲ | 22.0  ▲ | 20.7  ▲ | 22.1  ▲ | 28.8  ▲ | 21.9  ▲ | % | 11.5-15.5 |
| Platelets | 23  ▼ | 14  ▼ | 340 | 477  ▲ | 763  ▲ | 717  ▲ | 992  ▲ | 700  ▲ | 622  ▲ | 171 | 203 | 102  ▼ | 432 | 503  ▲ | x 10^3^/μL | 150-450 |
| Mean platelet volume | 10.8 ▲ | 9.8 | 9.4 | 11.3  ▲ | 11.5  ▲ | 11.0  ▲ | 10.2 | 11.3  ▲ | 11.3  ▲ | 8.2 | 8.8 | 9.1 | 9.4 | 10.5  ▲ | fL | 7.4 – 10.4 |
| Leukocytes | 2.3  ▼ | 2.29  ▼ | 14.4 ▲ | 36.0  ▲ | 144.6  ▲ | 127.6  ▲ | 65.5  ▲ | 28.5  ▲ | 31.6  ▲ | 4.3  ▼ | 6.7 | 5.8 | 18.4  ▲ | 36.4  ▲ | x 10^3^/μL | 4.5-11.0 |
| Segmented neutrophils | 22  ▼ | 34.0 ▼ | 43.0 | 54.0 | 58.0 | 67.0 | 71 |  | 79.0  ▲ |  | 52.0 | 43.0 | 65.0 | 65.0 | % | 40.0-75.0 |
| Lymphocytes | 76  ▲ | 63.7  ▲ | 21.0 | 13.0 | 8.0  ▼ | 3.0  ▼ | 10  ▼ |  | 12 |  | 31.0 | 46.0 | 22.0 | 9.0  ▼ | % | 12.0 -46.0 |
| Monocytes | 1.0 | 1.7 | 28.0 ▲ | 11.0 | 10.0 | 1.0 | 1.0 |  | 4.0 |  | 14.0  ▲ | 11.0 | 3.0 | 12.0 | % | 1.0-13.0 |
| Eosinophils | 0.0 | 0.0 | 0.0 | 3.0 | 2.0 | 1.0 | 3.0 |  | 3.0 |  | 3.0 | 0.0 | 9.0  ▲ | 2.0 | % | 0.0-7.0 |
| Basophiles | 1 | 0.6 | 0.0 | 2.0 | 3.0 | 3.0 | 4.0  ▲ |  | 1.0 |  | 0.0 | 0.0 | 1.0 | 6.0  ▲ | % | 0.0 -3.0 |
| Neutrophils in band | 0 | 1 | 2 | 4 | 1 | 0 | 0 |  | 0 |  | 0 | 0 | 0 | 2 | % | 0 – 4 |
| Absolute neutrophils |  | 0.8 | 6.2 | 19.4  ▲ | 83.9  ▲ | 85.5  ▲ | 49.83  ▲ |  | 25.0  ▲ |  | 3.5 | 2.5 | 12  ▲ | 23.7  ▲ | x 10^3^/μL | 1.8 -7.0 |
| Absolute lymphocytes |  | 1.5 | 3.0 | 4.7  ▲ | 11.6  ▲ | 3.8 | 7.92 |  | 3.8 |  | 2.1 | 2.7 | 4.0 | 3.3 | x 10^3^/μL | 1.2 -4.0 |
| Absolute Monocytes |  | 0.0 | 4.0  ▲ | 4.0  ▲ | 14.5  ▲ | 1.3  ▲ | 3.87  ▲ |  | 1.3  ▲ |  | 0.9  ▲ | 0.6 | 0.6 | 4.4  ▲ | x 10^3^/μL | 0.0 -0.8 |
| Absolute Eosinophils |  | 0.0 | 0.0 | 1.1  ▲ | 2.10  ▲ | 1.3  ▲ | 2.65 ▲ |  | 0.9  ▲ |  | 0.2 | 0.0 | 1.7  ▲ | 0.7  ▲ | x 10^3^/μL | 0.0 -0.5 |
| Absolute Basophiles |  | 0.0 | 0.0 | 0.7  ▲ | 4.3  ▲ | 3.8  ▲ | 1.24  ▲ |  | 0.3  ▲ |  | 0.0 | 0.0 | 0.2 | 2.2  ▲ | x 10^3^/μL | 0.0 -0.2 |
| Absolute bands |  | 0.0 | 0.3 | 1.4  ▲ | 1.4  ▲ | 0.0 | 0.0 |  | 0.0 |  | 0.0 | 0.0 | 0.0 | 0.7 | x 10^3^/μL | 0.0 – 0.7 |
| MORPHOLOGY |  |  |  |  |  |  |  |  |  |  |  |  |  |  |  |  |
| Anisocytosis | + | + | + | + |  | + |  |  | + |  | + | + | + | + |  |  |
| Erythroblasts/100 leukocytes |  |  | 5 | 2 | 3 |  |  |  |  |  |  |  |  | 4 |  |  |
| Metamyelocytes |  |  | 6 | 8 | 2 | 13 |  |  | 1 |  |  |  |  | 4 | % |  |
| Promyelocytes |  |  |  |  | 2 | 2 |  |  |  |  |  |  |  |  | % |  |
| Myelocytes |  |  |  | 5 | 14 | 11 |  |  |  |  |  |  |  |  | % |  |
| Macrocytes | + | + | + | + |  | + |  |  | + |  | + | + | + | + |  |  |
| Microcytes |  |  | + | + |  | + |  |  |  |  | + |  | + | + |  |  |
| Poikilocytes | + | + |  |  |  | + |  |  |  |  |  |  |  |  |  |  |
| Dacryocytes |  |  | + | + |  |  |  |  |  |  |  |  | + | + |  |  |
| Ovalocytes |  |  | + | + |  |  |  |  |  |  | + | + |  | + |  |  |
| Stomatocytes |  |  | + | + |  |  |  |  |  |  |  |  |  |  |  |  |
| Schistocytes |  |  |  | + |  | + |  |  | + |  |  |  |  | + |  |  |
| Acanthocytes |  |  |  |  |  |  |  |  |  |  | + |  |  |  |  |  |
| Target cells |  |  | + | + |  | + |  |  | + |  | + | + | + | + |  |  |
| Hypochromia |  |  |  |  |  | + |  |  |  |  |  |  |  | + |  |  |
| Anisochromia |  | + |  |  |  |  |  |  |  |  |  |  |  |  |  |  |
| Crenated erythrocytes |  |  |  |  |  |  |  |  | + |  | + |  |  |  |  |  |
| Erythrocytes with diffuse basophilia |  | + |  |  |  | + |  |  |  |  |  |  |  |  |  |  |
| Howell-jolly bodies |  | + |  |  |  |  |  |  |  |  |  |  |  |  |  |  |
| Helmet cells |  |  | + |  |  |  |  |  |  |  |  |  |  |  |  |  |
| Rouleaux |  | + |  | + |  |  |  |  |  |  |  |  |  |  |  |  |
| Giant and granular platelets |  |  |  | + |  | + |  |  | + |  |  | + |  |  |  |  |
| Prothrombin time  %  INR |  |  |  |  |  |  |  |  |  |  | 9.8  171.5  0.90 |  |  |  | s  % | 9.0 -12.0 s  0.50 –1.90 |
| Serum glucose |  |  |  |  | 116  ▲ | 78 |  | 114  ▲ |  |  | 92 |  |  |  | mg/dL | 72-100 |
| Serum urea nitrogen |  |  |  |  | 25.3  ▲ | 22.4  ▲ |  | 61.1  ▲ |  |  | 31.9  ▲ |  |  |  | mg/dL | 8.0 -20.0 |
| Creatinine |  |  |  |  | 1.56  ▲ | 1.45  ▲ |  | 1.28  ▲ |  |  | 1.35  ▲ |  |  | 1.75  ▲ | mg/dL | 0.44 –1.03 |
| Uric acid |  |  |  |  | 10.0  ▲ | 6.7 |  |  |  |  | 6.9 |  |  |  | mg/dL | 2.6-8.0 |
| Sodium |  |  |  |  | 139 | 140 |  | 142 |  |  | 140 |  |  |  | mmol/L | 136 - 144 |
| Potassium |  |  |  |  | 4.75 | 3.66 |  | 5.19  ▲ |  |  | 5.45  ▲ |  |  |  | mmol/L | 3.60- 5.10 |
| Serum chlorine |  |  |  |  | 112  ▲ | 114  ▲ |  | 112  ▲ |  |  | 112  ▲ |  |  |  | mmol/L | 101-111 |
| CO_2_ carbon dioxide |  |  |  |  | 17.2  ▼ | 15.5  ▼ |  | 23.4 |  |  | 19.3  ▼ |  |  |  | mmol/L | 22.0 –32.0 |
| Serum calcium |  |  |  |  | 9.8 | 7.7  ▼ |  | 9.5 |  |  | 9.6 |  |  |  | mg/dL | 8.9 – 10.3 |
| Corrected calcium (albumin) |  |  |  |  | 10.7  ▲ | 9.3 |  | 10.3 |  |  |  |  |  |  | mg/dL | 8.9 – 10.3 |
| Phosphorus serum |  |  |  |  | 4.3 | 3.9 |  | 5.4  ▲ |  |  | 4.3 |  |  |  | mg/dL | 2.4 – 4.7 |
| Magnesium |  |  |  |  | 1.64  ▼ | 1.12  ▼ |  | 1.49  ▼ |  |  | 1.91  ▼ |  |  |  | mg/dL | 1.80 –2.50 |
| Triglycerides |  |  |  |  |  | 89 |  |  |  |  | 95 |  |  |  | mg/dL | 10 - 149 |
| Cholesterol |  |  |  |  |  | 124  ▼ |  |  |  |  | 238  ▲ |  |  |  | mg/dL | 150 -199 |
| Cholesterol, HDL fraction |  |  |  |  |  | 37  ▼ |  |  |  |  | 64  ▲ |  |  |  | mg/dL | 40 – 60 |
| Cholesterol, non HDL |  |  |  |  |  | 86 |  |  |  |  | 175 |  |  |  | mg/dL |  |
| Cholesterol, LDL fraction |  |  |  |  |  | 68 |  |  |  |  | 156 |  |  |  | mg/dL |  |
| Total serum proteins |  |  |  |  | 6.3 |  |  |  |  |  | 7.0 |  |  |  | g/dL | 6.1 -7.9 |
| Albumin |  |  |  |  | 3.0  ▼ | 2.0 ▼ |  | 3.0  ▼ |  |  | 3.7 |  |  |  | g/dL | 3.5 – 4.8 |
| Globulins |  |  |  |  | 3.3 |  |  |  |  |  | 3.4 |  |  |  | g/dL | 2.3 -3.8 |
| Albumin/globulins ratio |  |  |  |  | 0.9  ▼ |  |  |  |  |  | 1.1 |  |  |  |  | 1.0 -2.2 |
| Total bilirubin |  |  |  |  | 0.32  ▼ |  |  |  |  |  | 0.34  ▼ |  |  |  | mg/dL | 0.40 -1.50 |
| Direct bilirubin |  |  |  |  | < 0.1 |  |  |  |  |  | < 0.1 |  |  |  | mg/dL | 0.0 – 0.30 |
| Alanine Aminotransferase |  |  |  |  | 15 |  |  |  |  |  | 13  ▼ |  |  |  | U/L | 14 – 54 |
| Aspartate aminotransferase |  |  |  |  | 19 |  |  |  |  |  | 19 |  |  |  | U/L | 15-41 |
| Alkaline phosphatase |  |  |  |  | 127  ▲ |  |  |  |  |  | 126  ▲ |  |  |  | U/L | 32 – 91 |
| Gamaglutamil transpeptidase |  |  |  |  | 32 |  |  |  |  |  | 20 |  |  |  | U/L | 7 – 50 |
| Lactic dehydrogenase |  |  |  |  | 508  ▲ |  |  |  |  |  | 143 |  |  |  | U/L | 98 – 192 |
| Creatine phosphokinase (CPK) |  |  |  |  |  |  |  |  |  |  | 58 |  |  |  | U/L | 38 -234 |
| Serum iron |  |  |  |  | 9  ▼ |  |  | 28 |  |  | 135 |  |  |  | μg/dL | 28 – 170 |
| Iron fixing capacity |  |  |  |  | 298 |  |  | 196  ▼ |  |  | 474 |  |  |  | μg/dL | 261-478 |
| % Transferrin saturation |  |  |  |  | 3  ▼ |  |  | 14  ▼ |  |  | 29 |  |  |  | % | 17- 47 |
| C reactive protein |  |  |  |  |  | 191.9  ▲ |  |  |  |  |  |  |  |  | mg/dL | 0.0 -7.4 |
| procalcitonin |  |  |  |  |  | 0.52  ▲ |  |  |  |  |  |  |  |  | ng/Ml | 0 – 0.06 |
| In grey the analysis during leptospirosis ▲; Level above the reference interval, ▼; level below the reference interval, +; Moderate. | | | | | | | | | | | | | | | | |

Supplementary Table 2. Results of the Microscopic agglutination test (MAT).

| *Leptospira* Serovar | Sera samples | |
| --- | --- | --- |
|  | first | second |
| Autumnalis, Akiyami A | - | - |
| Bataviae, Van Tienen | 1/10 | 1/80 |
| Bratislava, Jez Bratislava | 1/320 | 1/320 |
| Canicola, Hond Utech IV | 1/20 | 1/40 |
| Celledoni, Celledoni | - | - |
| Grippotyphosa, Moska V | 1/80 | 1/40 |
| Hardjo, Hardjoprajitno | 1/80 | 1/40 |
| Icterohaemorrhagiae RGA | - | - |
| Pomona, Pomona | 1/10 |  |
| Pyrogenes, Salinem | 1/160 | 1/80 |
| Tarassovi, Parepelicin | - | - |
| Wolffi, 3705 | - | - |
| Mini, Georgia LT117 | - | - |

Supplementary Methodology document.

***Microagglutination test* (MAT)**

The blood samples tested by MAT were separated from each other by ten days. The first sample was taken on admission, on October the 10^th^, 2013, and the second sample was taken on October the 20^th^, 2013. The recommended interval between both samples is 15 days; in this case, our reference was the admission date as the most probable onset time.

***Leptospira culture and isolation***

The hamster inoculated with the patient’s sample developed sublethal leptospirosis with mild symptoms and managed to survive. It was sacrificed and its blood and tissues; liver, kidney and spleen, were inoculated into EMJH medium to retrieve *Leptospira*. They were also incubated at 30 °C and examined periodically by dark-field microscopy. In this medium, we support the *Leptospira* survival for about a month, but we could not achieve the conditions to allow the culture to flourish. The *IS*1500 PCR assisted the following of the bacteria subcultures.

The Bact/Alert FA medium is not a specific culture media for *Leptospira*. This medium is not used for isolation, neither use for further maintenance of *Leptospira,* as it needs specific nutrients to thrive.

All results are summarised in the following Supplementary table 3.

Supplementary table 3. Results summary

| **SAMPLE** | **MAT** | ***IS*1500 PCR** | **EMJH MEDIUM** |
| --- | --- | --- | --- |
| first blood sample | - positive | - Positive | - no isolation |
| second blood sample | - positive | - positive |  |
| hamster blood pre-inoculation of patients’ blood^&^ | - negative |  |  |
| BacT/Alert culture |  | - Positive |  |
| hamster blood |  | - Positive | - no isolation |
| hamster liver |  | - Positive | - no isolation |
| hamster spleen |  | - Positive | - no isolation |
| hamster kidney |  | - Positive | - no isolation |

^&^the animal was screened before inoculation according to the hamster model to study leptospirosis.

***Leptospira* genus-specific PCR.**

The reaction was carried out in a final volume of 50 μL with 0.5 μg of DNA, 1.2 μM of primer L737 (5´-GACCCGAAGCCTGTCGAG-3’) and primer L1218 (5’- GCCATGCTTAGTCCCGATTAC-3’), 0.2 mM of each dNTP, 10 μL of 5X GoTaq Flexi Buffer, 3 mM MgCl_2_, 0.5 mg/mL of bovine serum albumin (BSA), and 2.5 U GoTaq (Promega, Madison, Wisconsin, USA). The PCR conditions were an initial denaturation at 94 °C for 5 min, followed by 45 cycles of denaturation at 94 °C for 15 s, annealing at 59 °C for 40 s, and an extension at 74 °C for 1 min and 20 s, and a final extension at 74 °C for 10 min [10].

***Leptospira* pathogenic-species PCR.**

The primers P1 (5’- TCGCTGAAATRGGWGTTCGT-3’) and M16 (5’-CGCCTGGYTCMCCGATT-3’) amplifies a fragment of 660 bp [11]. The reaction was performed in a final volume of 50 μL with 0.5 μg of DNA, 1 mM of each primer, 0.2 mM of each dNTP, 10 μL of 5X GoTaq Flexi Buffer, 3 mM MgCl_2_, 0.5 mg/mL of BSA, 1% Triton X-100, and 2.5 U GoTaq (Promega, Madison, Wisconsin, USA). The PCR protocol was an initial denaturation at 94 °C for 5 min, followed by 40 cycles of denaturation at 94 °C for 30 s, annealing at 60 °C for 45 s, and extension at 74 °C for 1 min with an increment of 5 s/cycle, and a final extension at 74 °C for 10 min. The positive DNA control was genomic DNA of serovar Pomona, and a non-template control was performed.

**Genus identification**

Three reactions were performed in final volume of 50 μL with 0.4 μg of DNA, 1 mM of each primer, 0.2 mM of each dNTP, 5 μL of 10 X HiFi Buffer, 1.5 mM MgCl_2_, and 5 U Platinum Taq DNA Polymerase High Fidelity (Invitrogen, Carlsbad, CA). The amplification conditions were an initial denaturation for 5 min at 95 °C, followed by 40 cycles of denaturation at 95 °C for 45 s, annealing at 55 °C for 1 min, and extension at 72 °C for 2 min, with a single final extension at 72 °C for 7 min [12]. After electrophoresis, the clean agarose gel band was excised from the gel, and the PCR product was purified with the QIAquick purification kit (Qiagen, Ventura, CA, USA), according to the manufacturer’s instructions. The DNA was quantified in an Epoch microplate spectrophotometer (Biotech). A vial with 100 ng of the PCR purified product in a final volume of 16 μL, and 10 pmol of each primer were sent to the *Instituto de Biotecnología* for sequencing in both directions. Each sequence was visualised in the Bioedit software (freely available at <http://www.mbio.ncsu.edu/BioEdit/bioedit.html>), and the consensus sequence has homology and identity with other *Leptospira* sequences according to the BLAST with sequences of the NCBI Transcript Reference Sequences database as shown in the following Supplementary Table 4.

Supplementary Table 4.- Results of BLAST with Reference Sequences.

| Accession no | Identity % | Alignment length | Mismatches | Gap opens | q.start | q. end | s. start | s. end | E Value | Bit score |
| --- | --- | --- | --- | --- | --- | --- | --- | --- | --- | --- |
| NR_043050.1 | 99.364 | 1415 | 9 | 0 | 18 | 1432 | 1431 | 17 | 0.0 | 2567 |
| NR_029361.1 | 99.928 | 1390 | 0 | 1 | 30 | 1418 | 1396 | 7 | 0.0 | 2560 |
| NR_043259.1 | 99.293 | 1415 | 10 | 0 | 18 | 1432 | 1431 | 17 | 0.0 | 2558 |
| NR_043047.1 | 99.011 | 1416 | 13 | 1 | 18 | 1432 | 1432 | 17 | 0.0 | 2538 |
| NR_043048.1 | 98.799 | 1415 | 17 | 0 | 18 | 1432 | 1431 | 17 | 0.0 | 2521 |
| NR_119300.1 | 98.785 | 1399 | 14 | 3 | 34 | 1432 | 1410 | 15 | 0.0 | 2486 |
| NR_114968.1 | 100.000 | 1318 | 0 | 0 | 111 | 1428 | 1318 | 1 | 0.0 | 2435 |
| NR_114969.1 | 99.241 | 1318 | 10 | 0 | 111 | 1428 | 1318 | 1 | 0.0 | 2379 |
| NR_041544.1 | 99.005 | 1307 | 13 | 0 | 126 | 1432 | 1331 | 25 | 0.0 | 2342 |
| NR_118435.1 | 99.073 | 1295 | 12 | 0 | 138 | 1432 | 1318 | 24 | 0.0 | 2326 |
| NR_134067.1 | 98.849 | 1303 | 15 | 0 | 130 | 1432 | 1313 | 11 | 0.0 | 2324 |
| NR_115296.1 | 95.124 | 1415 | 69 | 0 | 18 | 1432 | 1431 | 17 | 0.0 | 2231 |
| NR_043049.1 | 95.124 | 1415 | 69 | 0 | 18 | 1432 | 1431 | 17 | 0.0 | 2231 |
| NR_043200.1 | 94.912 | 1415 | 72 | 0 | 18 | 1432 | 1431 | 17 | 0.0 | 2215 |
| NR_115234.1 | 100.000 | 1181 | 0 | 0 | 221 | 1401 | 1181 | 1 | 0.0 | 2182 |
| NR_115235.1 | 98.899 | 1181 | 13 | 0 | 221 | 1401 | 1181 | 1 | 0.0 | 2109 |
| NR_114336.1 | 89.400 | 1434 | 131 | 16 | 5 | 1432 | 1443 | 25 | 0.0 | 1786 |
| NR_115297.1 | 89.231 | 1430 | 115 | 29 | 18 | 1432 | 1422 | 17 | 0.0 | 1751 |
| NR_043046.1 | 89.146 | 1428 | 120 | 26 | 18 | 1432 | 1422 | 17 | 0.0 | 1748 |
| NR_043043.1 | 89.153 | 1429 | 118 | 28 | 18 | 1432 | 1422 | 17 | 0.0 | 1748 |
| NR_115294.1 | 89.153 | 1429 | 118 | 28 | 18 | 1432 | 1422 | 17 | 0.0 | 1746 |
| NR_115293.1 | 88.936 | 1428 | 123 | 26 | 18 | 1432 | 1422 | 17 | 0.0 | 1740 |
| M88721.1 | 88.991 | 1417 | 121 | 26 | 29 | 1432 | 1462 | 68 | 0.0 | 1727 |
| NR_043045.1 | 88.725 | 1428 | 126 | 26 | 18 | 1432 | 1422 | 17 | 0.0 | 1724 |
| M71241.1 | 97.849 | 837 | 18 | 0 | 26 | 862 | 1321 | 485 | 0.0 | 1478 |
| M71241.1 | 97.964 | 442 | 9 | 0 | 991 | 1432 | 484 | 43 | 0.0 | 780 |
